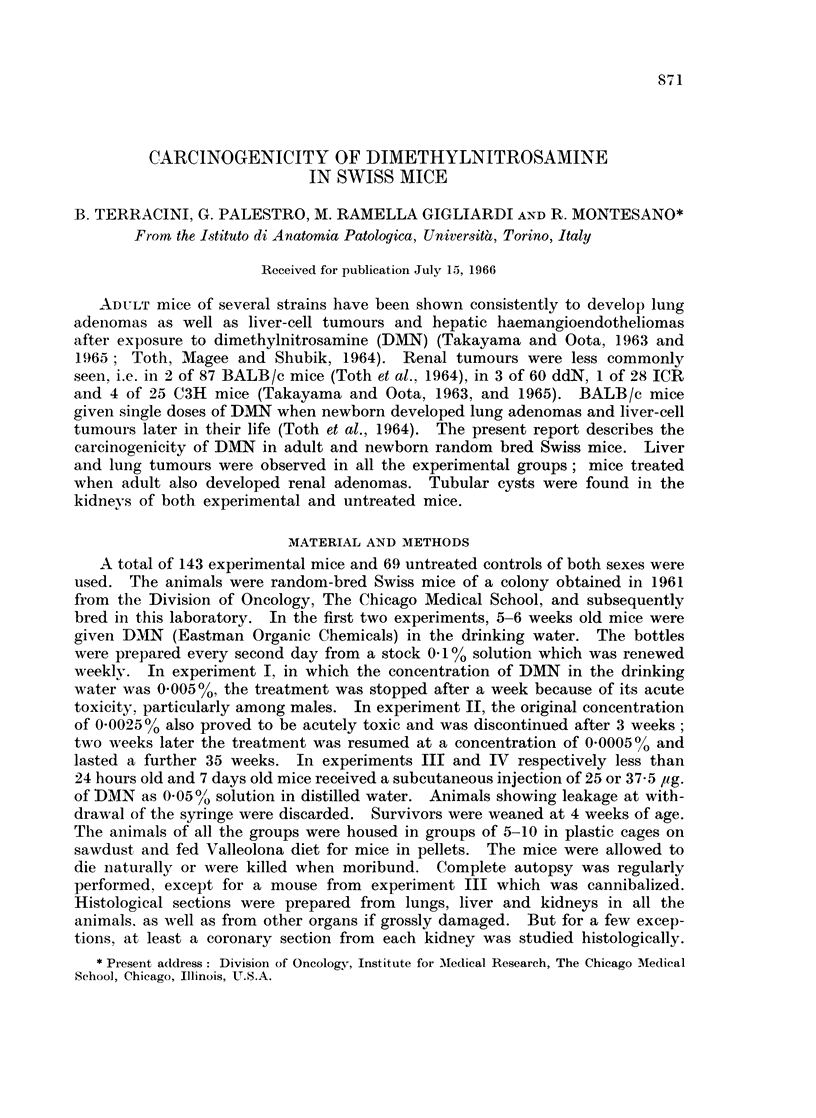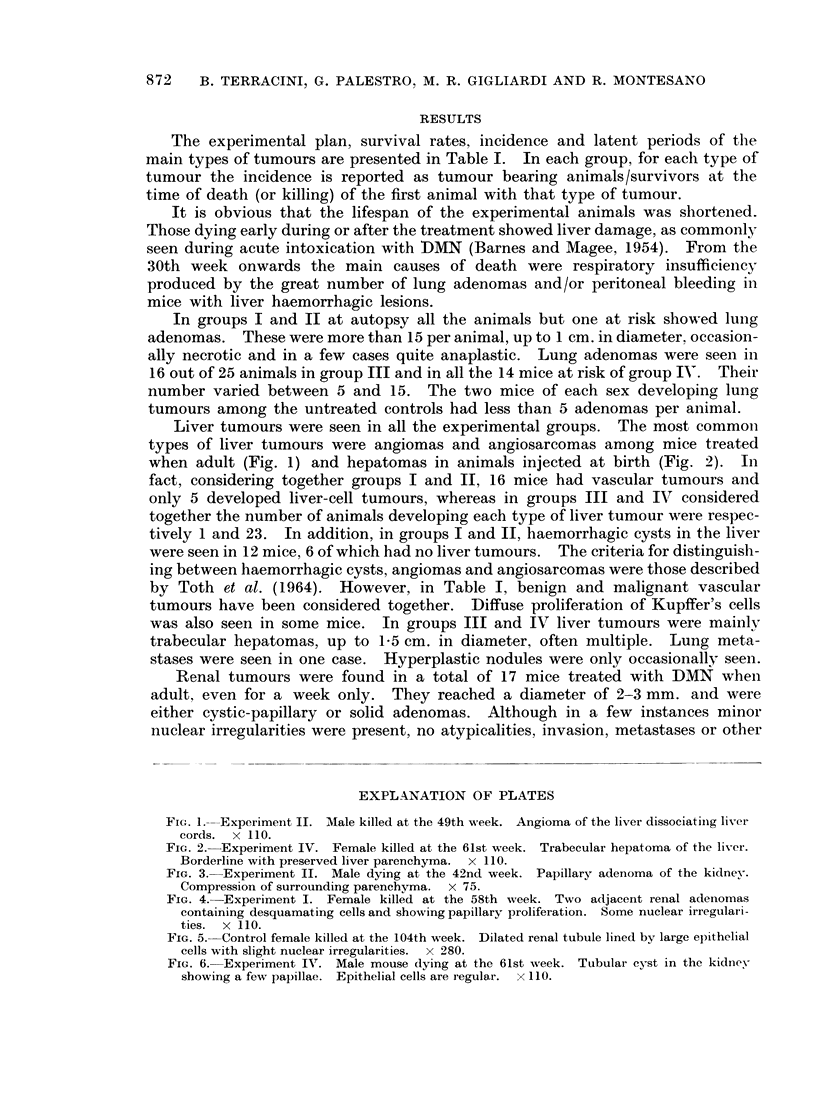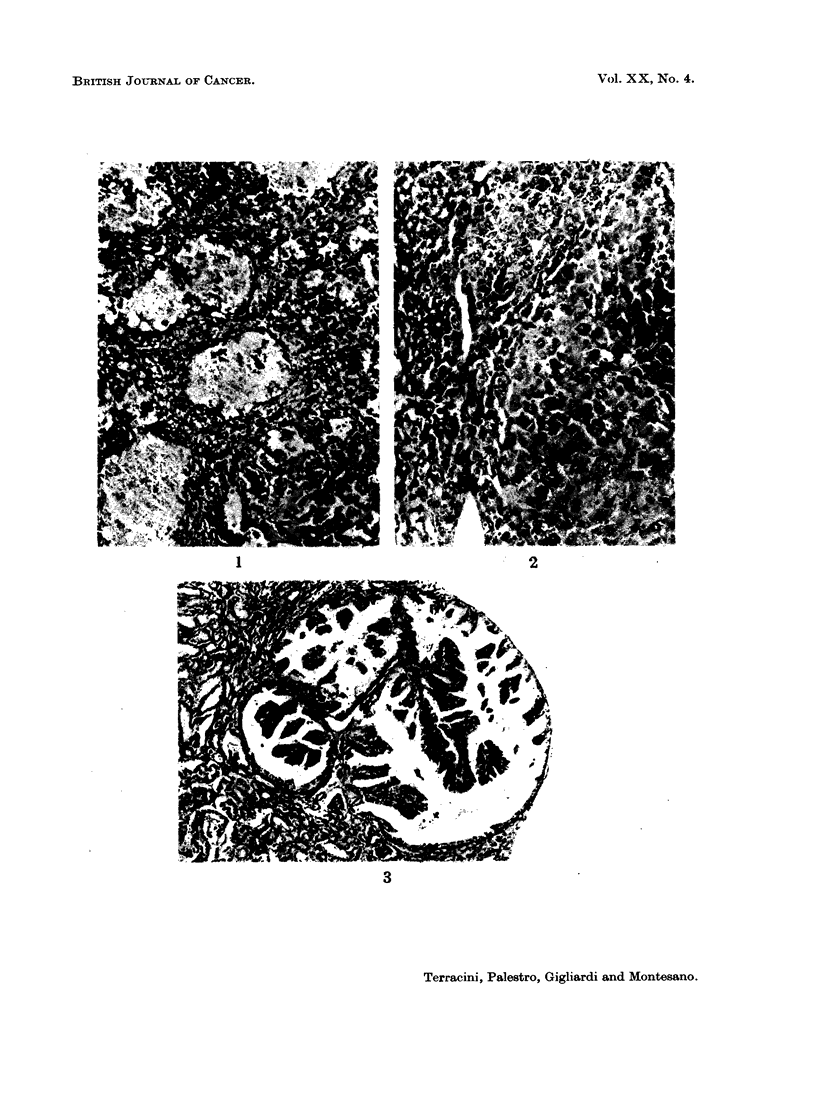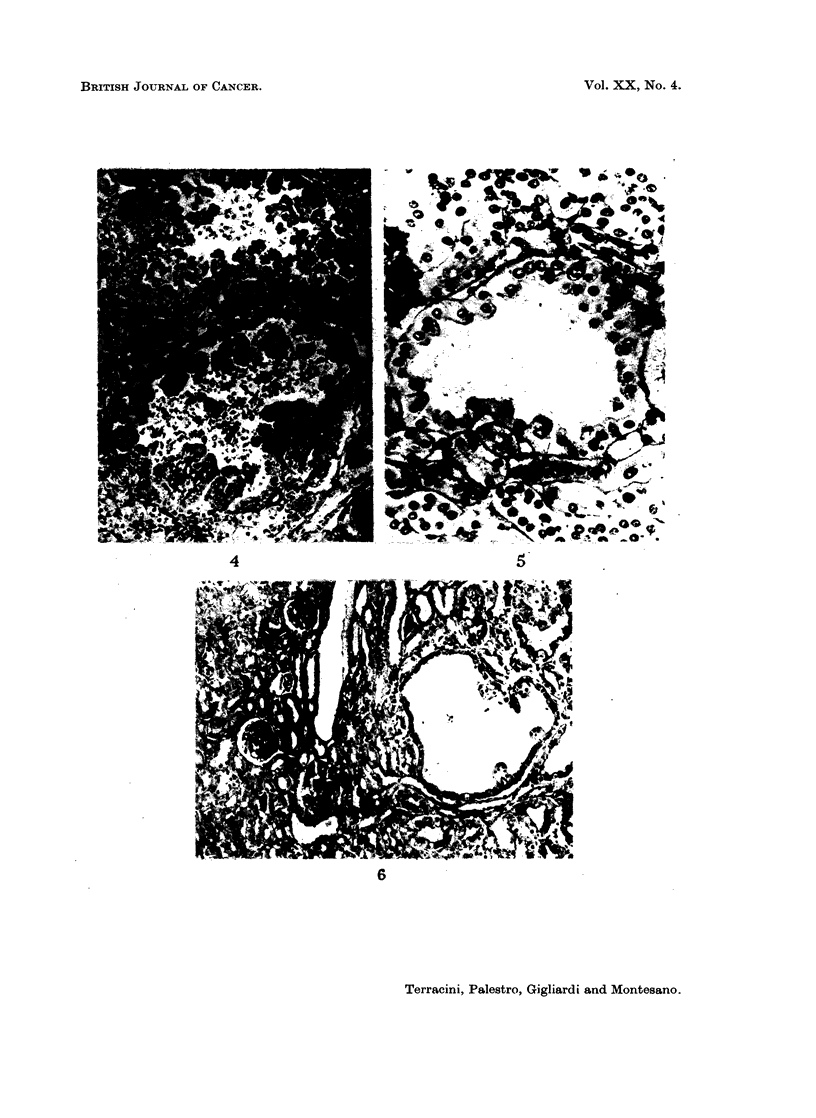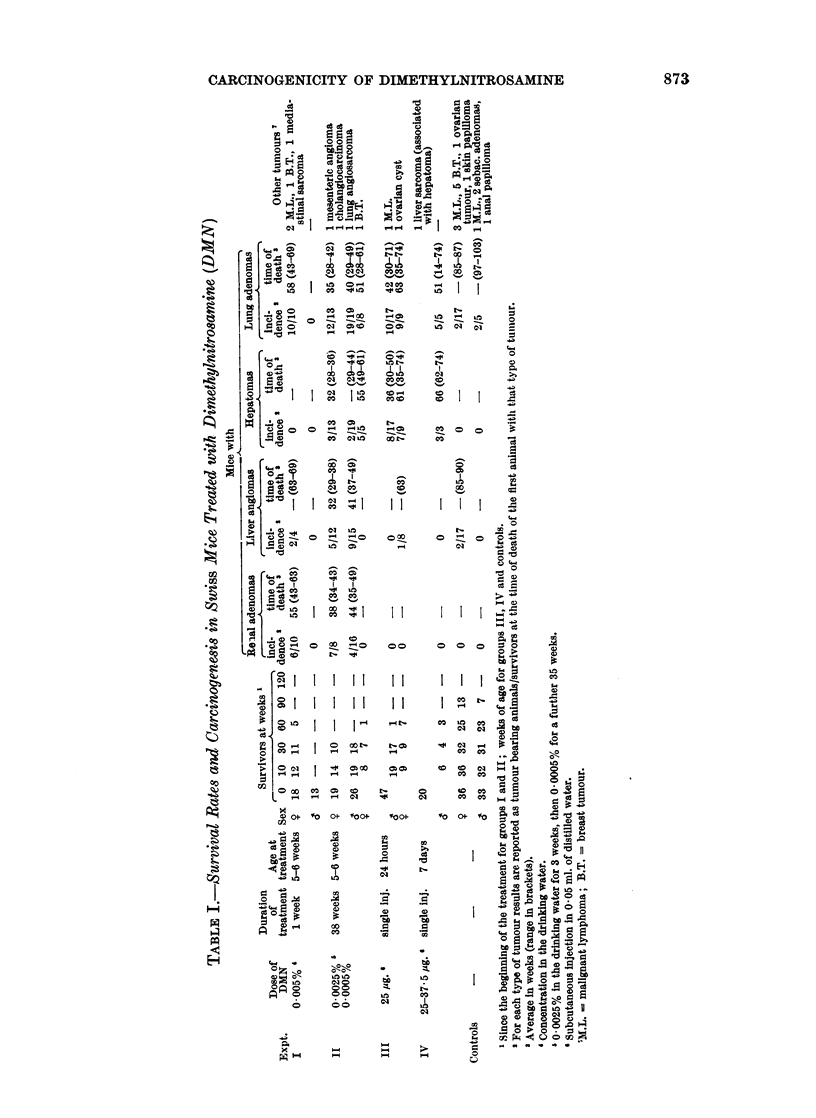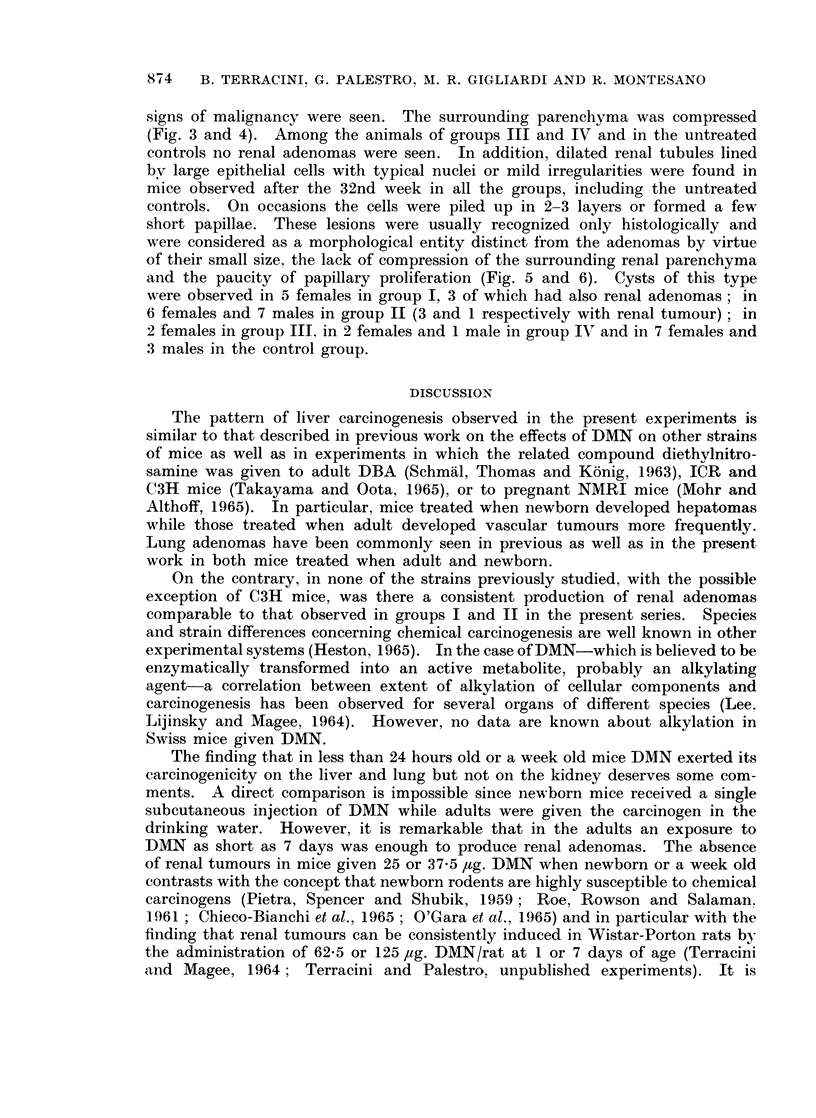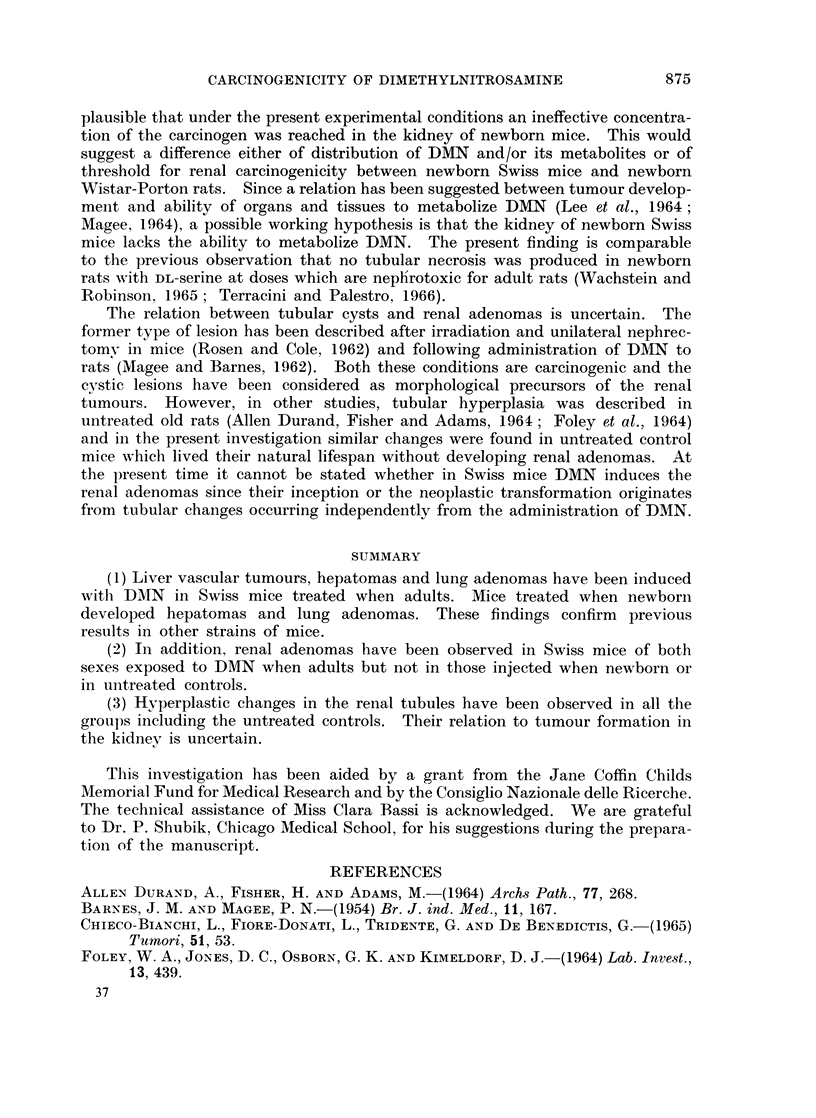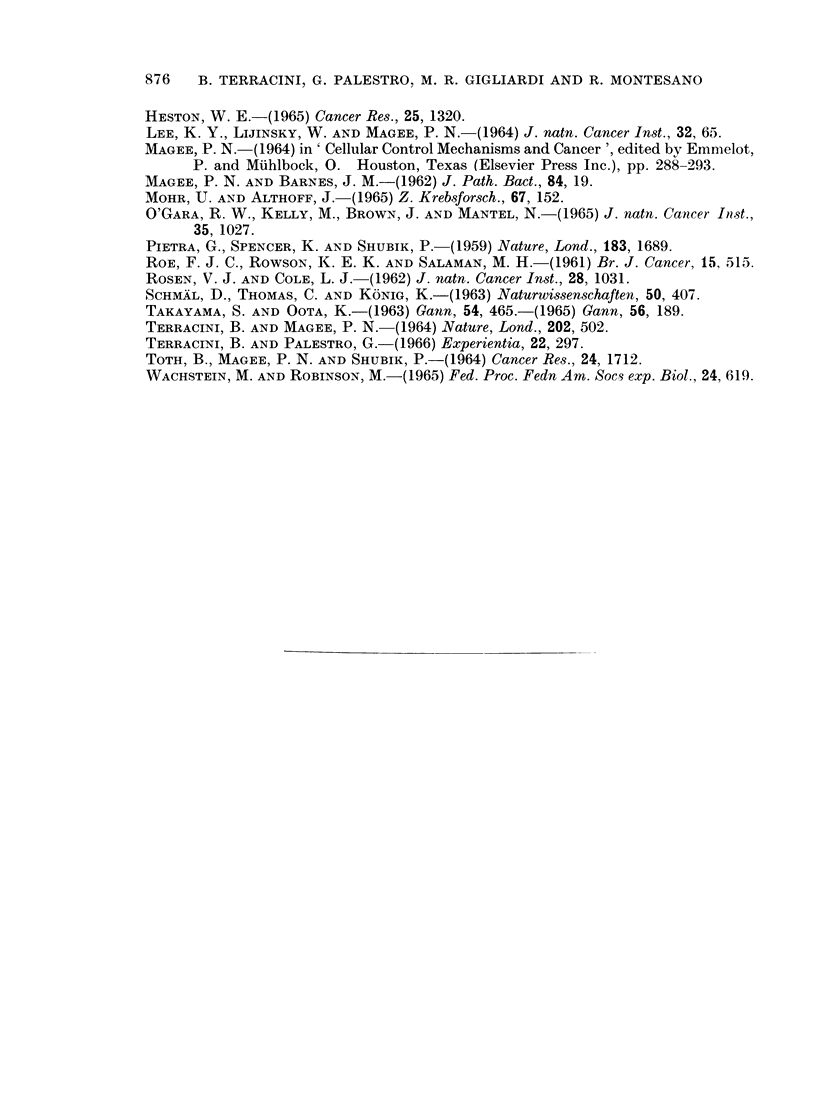# Carcinogenicity of dimethylnitrosamine in Swiss mice.

**DOI:** 10.1038/bjc.1966.100

**Published:** 1966-12

**Authors:** B. Terracini, G. Palestro, M. R. Gigliardi, G. Montesano, R. Montesano

## Abstract

**Images:**


### CARCINOGENICITY OF DIMETHYLNITROSAMINE IN SWISS MICE

B. TERRACINI, G. PALESTRO, M. RAMELLA GIGLIARDI AND R. MONTESANO*

*From the Istituto di Anatomia Patologica, Università, Torino, Italy*

Received for publication July 15, 1966

ADULT mice of several strains have been shown consistently to develop lung adenomas as well as liver-cell tumours and hepatic haemangioendotheliomas after exposure to dimethylnitrosamine (DMN) (Takayama and Oota, 1963 and 1965 ; Toth, Magee and Shubik, 1964). Renal tumours were less commonly seen, i.e. in 2 of 87 BALB/c mice (Toth *et al.*, 1964), in 3 of 60 ddN, 1 of 28 ICR and 4 of 25 C3H mice (Takayama and Oota, 1963, and 1965). BALB/c mice given single doses of DMN when newborn developed lung adenomas and liver-cell tumours later in their life (Toth *et al.*, 1964). The present report describes the carcinogenicity of DMN in adult and newborn random bred Swiss mice. Liver and lung tumours were observed in all the experimental groups ; mice treated when adult also developed renal adenomas. Tubular cysts were found in the kidneys of both experimental and untreated mice.

### MATERIAL AND METHODS

A total of 143 experimental mice and 69 untreated controls of both sexes were used. The animals were random-bred Swiss mice of a colony obtained in 1961 from the Division of Oncology, The Chicago Medical School, and subsequently bred in this laboratory. In the first two experiments, 5–6 weeks old mice were given DMN (Eastman Organic Chemicals) in the drinking water. The bottles were prepared every second day from a stock 0·1% solution which was renewed weekly. In experiment I, in which the concentration of DMN in the drinking water was 0·005%, the treatment was stopped after a week because of its acute toxicity, particularly among males. In experiment II, the original concentration of 0·0025% also proved to be acutely toxic and was discontinued after 3 weeks ; two weeks later the treatment was resumed at a concentration of 0·0005% and lasted a further 35 weeks. In experiments III and IV respectively less than 24 hours old and 7 days old mice received a subcutaneous injection of 25 or 37·5 $\mu$g. of DMN as 0·05% solution in distilled water. Animals showing leakage at withdrawal of the syringe were discarded. Survivors were weaned at 4 weeks of age. The animals of all the groups were housed in groups of 5–10 in plastic cages on sawdust and fed Valleolona diet for mice in pellets. The mice were allowed to die naturally or were killed when moribund. Complete autopsy was regularly performed, except for a mouse from experiment III which was cannibalized. Histological sections were prepared from lungs, liver and kidneys in all the animals, as well as from other organs if grossly damaged. But for a few exceptions, at least a coronary section from each kidney was studied histologically.

* Present address : Division of Oncology, Institute for Medical Research, The Chicago Medical School, Chicago, Illinois, U.S.A.

### RESULTS

The experimental plan, survival rates, incidence and latent periods of the main types of tumours are presented in Table I. In each group, for each type of tumour the incidence is reported as tumour bearing animals/survivors at the time of death (or killing) of the first animal with that type of tumour.

It is obvious that the lifespan of the experimental animals was shortened. Those dying early during or after the treatment showed liver damage, as commonly seen during acute intoxication with DMN (Barnes and Magee, 1954). From the 30th week onwards the main causes of death were respiratory insufficiency produced by the great number of lung adenomas and/or peritoneal bleeding in mice with liver haemorrhagic lesions.

In groups I and II at autopsy all the animals but one at risk showed lung adenomas. These were more than 15 per animal, up to 1 cm. in diameter, occasionally necrotic and in a few cases quite anaplastic. Lung adenomas were seen in 16 out of 25 animals in group III and in all the 14 mice at risk of group IV. Their number varied between 5 and 15. The two mice of each sex developing lung tumours among the untreated controls had less than 5 adenomas per animal.

Liver tumours were seen in all the experimental groups. The most common types of liver tumours were angiomas and angiosarcomas among mice treated when adult (Fig. 1) and hepatomas in animals injected at birth (Fig. 2). In fact, considering together groups I and II, 16 mice had vascular tumours and only 5 developed liver-cell tumours, whereas in groups III and IV considered together the number of animals developing each type of liver tumour were respectively 1 and 23. In addition, in groups I and II, haemorrhagic cysts in the liver were seen in 12 mice, 6 of which had no liver tumours. The criteria for distinguishing between haemorrhagic cysts, angiomas and angiosarcomas were those described by Toth *et al.* (1964). However, in Table I, benign and malignant vascular tumours have been considered together. Diffuse proliferation of Kupffer's cells was also seen in some mice. In groups III and IV liver tumours were mainly trabecular hepatomas, up to 1·5 cm. in diameter, often multiple. Lung metastases were seen in one case. Hyperplastic nodules were only occasionally seen.

Renal tumours were found in a total of 17 mice treated with DMN when adult, even for a week only. They reached a diameter of 2–3 mm. and were either cystic-papillary or solid adenomas. Although in a few instances minor nuclear irregularities were present, no atypicalities, invasion, metastases or other

---

### EXPLANATION OF PLATES

FIG. 1.—Experiment II. Male killed at the 49th week. Angioma of the liver dissociating liver cords. × 110.

FIG. 2.—Experiment IV. Female killed at the 61st week. Trabecular hepatoma of the liver. Borderline with preserved liver parenchyma. × 110.

FIG. 3.—Experiment II. Male dying at the 42nd week. Papillary adenoma of the kidney. Compression of surrounding parenchyma. × 75.

FIG. 4.—Experiment I. Female killed at the 58th week. Two adjacent renal adenomas containing desquamating cells and showing papillary proliferation. Some nuclear irregularities. × 110.

FIG. 5.—Control female killed at the 104th week. Dilated renal tubule lined by large epithelial cells with slight nuclear irregularities. × 280.

FIG. 6.—Experiment IV. Male mouse dying at the 61st week. Tubular cyst in the kidney showing a few papillae. Epithelial cells are regular. ×110.

1 2

3

Terracini, Palestro, Gigliardi and Montesano.

signs of malignancy were seen. The surrounding parenchyma was compressed (Fig. 3 and 4). Among the animals of groups III and IV and in the untreated controls no renal adenomas were seen. In addition, dilated renal tubules lined by large epithelial cells with typical nuclei or mild irregularities were found in mice observed after the 32nd week in all the groups, including the untreated controls. On occasions the cells were piled up in 2–3 layers or formed a few short papillae. These lesions were usually recognized only histologically and were considered as a morphological entity distinct from the adenomas by virtue of their small size, the lack of compression of the surrounding renal parenchyma and the paucity of papillary proliferation (Fig. 5 and 6). Cysts of this type were observed in 5 females in group I, 3 of which had also renal adenomas ; in 6 females and 7 males in group II (3 and 1 respectively with renal tumour) ; in 2 females in group III, in 2 females and 1 male in group IV and in 7 females and 3 males in the control group.

### DISCUSSION

The pattern of liver carcinogenesis observed in the present experiments is similar to that described in previous work on the effects of DMN on other strains of mice as well as in experiments in which the related compound diethylnitrosamine was given to adult DBA (Schmäl, Thomas and König, 1963), ICR and C3H mice (Takayama and Oota, 1965), or to pregnant NMRI mice (Mohr and Althoff, 1965). In particular, mice treated when newborn developed hepatomas while those treated when adult developed vascular tumours more frequently. Lung adenomas have been commonly seen in previous as well as in the present work in both mice treated when adult and newborn.

On the contrary, in none of the strains previously studied, with the possible exception of C3H mice, was there a consistent production of renal adenomas comparable to that observed in groups I and II in the present series. Species and strain differences concerning chemical carcinogenesis are well known in other experimental systems (Heston, 1965). In the case of DMN—which is believed to be enzymatically transformed into an active metabolite, probably an alkylating agent—a correlation between extent of alkylation of cellular components and carcinogenesis has been observed for several organs of different species (Lee, Lijinsky and Magee, 1964). However, no data are known about alkylation in Swiss mice given DMN.

The finding that in less than 24 hours old or a week old mice DMN exerted its carcinogenicity on the liver and lung but not on the kidney deserves some comments. A direct comparison is impossible since newborn mice received a single subcutaneous injection of DMN while adults were given the carcinogen in the drinking water. However, it is remarkable that in the adults an exposure to DMN as short as 7 days was enough to produce renal adenomas. The absence of renal tumours in mice given 25 or 37·5 $\mu$g. DMN when newborn or a week old contrasts with the concept that newborn rodents are highly susceptible to chemical carcinogens (Pietra, Spencer and Shubik, 1959 ; Roe, Rowson and Salaman, 1961 ; Chieco-Bianchi *et al.*, 1965 ; O'Gara *et al.*, 1965) and in particular with the finding that renal tumours can be consistently induced in Wistar-Porton rats by the administration of 62·5 or 125 $\mu$g. DMN/rat at 1 or 7 days of age (Terracini and Magee, 1964 ; Terracini and Palestro, unpublished experiments). It is plausible that under the present experimental conditions an ineffective concentration of the carcinogen was reached in the kidney of newborn mice. This would suggest a difference either of distribution of DMN and/or its metabolites or of threshold for renal carcinogenicity between newborn Swiss mice and newborn Wistar-Porton rats. Since a relation has been suggested between tumour development and ability of organs and tissues to metabolize DMN (Lee *et al.*, 1964; Magee, 1964), a possible working hypothesis is that the kidney of newborn Swiss mice lacks the ability to metabolize DMN. The present finding is comparable to the previous observation that no tubular necrosis was produced in newborn rats with DL-serine at doses which are nephrotoxic for adult rats (Wachstein and Robinson, 1965; Terracini and Palestro, 1966).

The relation between tubular cysts and renal adenomas is uncertain. The former type of lesion has been described after irradiation and unilateral nephrectomy in mice (Rosen and Cole, 1962) and following administration of DMN to rats (Magee and Barnes, 1962). Both these conditions are carcinogenic and the cystic lesions have been considered as morphological precursors of the renal tumours. However, in other studies, tubular hyperplasia was described in untreated old rats (Allen Durand, Fisher and Adams, 1964; Foley *et al.*, 1964) and in the present investigation similar changes were found in untreated control mice which lived their natural lifespan without developing renal adenomas. At the present time it cannot be stated whether in Swiss mice DMN induces the renal adenomas since their inception or the neoplastic transformation originates from tubular changes occurring independently from the administration of DMN.

SUMMARY

(1) Liver vascular tumours, hepatomas and lung adenomas have been induced with DMN in Swiss mice treated when adults. Mice treated when newborn developed hepatomas and lung adenomas. These findings confirm previous results in other strains of mice.

(2) In addition, renal adenomas have been observed in Swiss mice of both sexes exposed to DMN when adults but not in those injected when newborn or in untreated controls.

(3) Hyperplastic changes in the renal tubules have been observed in all the groups including the untreated controls. Their relation to tumour formation in the kidney is uncertain.

This investigation has been aided by a grant from the Jane Coffin Childs Memorial Fund for Medical Research and by the Consiglio Nazionale delle Ricerche. The technical assistance of Miss Clara Bassi is acknowledged. We are grateful to Dr. P. Shubik, Chicago Medical School, for his suggestions during the preparation of the manuscript.

### REFERENCES

Allen Durand, A., Fisher, H. and Adams, M.—(1964) *Archs Path.*, **77**, 268.

Barnes, J. M. and Magee, P. N.—(1954) *Br. J. ind. Med.*, **11**, 167.

Chieco-Bianchi, L., Fiore-Donati, L., Tridente, G. and De Benedictis, G.—(1965) *Tumori*, **51**, 53.

Foley, W. A., Jones, D. C., Osborn, G. K. and Kimeldorf, D. J.—(1964) *Lab. Invest.*, **13**, 439.

HESTON, W. E.—(1965) *Cancer Res.*, **25**, 1320.
LEE, K. Y., LIJINSKY, W. AND MAGEE, P. N.—(1964) *J. natn. Cancer Inst.*, **32**, 65.
MAGEE, P. N.—(1964) in ' Cellular Control Mechanisms and Cancer ', edited by Emmelot, P. and Mühlbock, O. Houston, Texas (Elsevier Press Inc.), pp. 288–293.
MAGEE, P. N. AND BARNES, J. M.—(1962) *J. Path. Bact.*, **84**, 19.
MOHR, U. AND ALTHOFF, J.—(1965) *Z. Krebsforsch.*, **67**, 152.
O'GARA, R. W., KELLY, M., BROWN, J. AND MANTEL, N.—(1965) *J. natn. Cancer Inst.*, **35**, 1027.
PIETRA, G., SPENCER, K. AND SHUBIK, P.—(1959) *Nature, Lond.*, **183**, 1689.
ROE, F. J. C., ROWSON, K. E. K. AND SALAMAN, M. H.—(1961) *Br. J. Cancer*, **15**, 515.
ROSEN, V. J. AND COLE, L. J.—(1962) *J. natn. Cancer Inst.*, **28**, 1031.
SCHMÄL, D., THOMAS, C. AND KÖNIG, K.—(1963) *Naturwissenschaften*, **50**, 407.
TAKAYAMA, S. AND OOTA, K.—(1963) *Gann*, **54**, 465.—(1965) *Gann*, **56**, 189.
TERRACINI, B. AND MAGEE, P. N.—(1964) *Nature, Lond.*, **202**, 502.
TERRACINI, B. AND PALESTRO, G.—(1966) *Experientia*, **22**, 297.
TOTH, B., MAGEE, P. N. AND SHUBIK, P.—(1964) *Cancer Res.*, **24**, 1712.
WACHSTEIN, M. AND ROBINSON, M.—(1965) *Fed. Proc. Fedn Am. Socs exp. Biol.*, **24**, 619.